# Losing psychiatric or psychological follow-up during the first COVID-19 confinement in Portugal: outcomes in mental health

**DOI:** 10.1192/j.eurpsy.2023.1239

**Published:** 2023-07-19

**Authors:** M. Gomes, P. Silva Moreira, S. Ferreira, B. Couto, M. Machado-Sousa, C. Raposo-Lima, N. Sousa, M. Picó-Pérez, P. Morgado

**Affiliations:** 1 Life and Health Sciences Research Institute (ICVS), School of Medicine, University of Minho, Braga; 2 ICVS/3B’s, PT Government Associate Laboratory, Braga/Guimarães; 3 Psychological Neuroscience Lab, CIPsi, School of Psychology, University of Minho, Braga, Portugal; 4 Departamento de Psicología Básica, Clínica y Psicobiología, Universitat Jaume I, Castelló de la Plana, Spain

## Abstract

**Introduction:**

The COVID-19 outbreak imposed several periods of lockdown to stop the pandemic, with a determinant impact on access to mental health services. In Portugal, the first State of Emergency was declared on the 18th of March 2020, with the obligation of mandatory confinement and circulation restriction. Restrictive measures were alleviated on the 2nd of May 2020.

**Objectives:**

We aimed to investigate the impact of the first confinement on the maintenance or loss of psychiatric and psychological follow-up. Also, we aimed to explore the outcomes in the mental health of losing psychiatric or psychological consultations.

**Methods:**

We conducted an online survey among the Portuguese population to evaluate demographic, clinical and mental health variables (STAI, DASS-21, PHQ, OCI-R, Quality of Life [QoL] and PSS). Individuals were invited to answer the survey at two timepoints: third week of March 2020 and third week of May 2020. Concerning the first timepoint, we used independent t-tests to compare the mental health variables in the individuals who loss and who did not lose consultations. Then, we evaluated the impact of losing consultations across time in those individuals who continued responding in the second timepoint, through a Linear Fixed Model. All the analyses were performed using JASP software.

**Results:**

From the total sample (n=2040), 334 individuals (84.4% female gender) had psychiatric and/or psychological consultations previously to the confinement. In March 2020, the individuals who maintained the consultations (35.0%) showed best mental health indicators in the QoL Self Evaluation (p=0.002), QoL Satisfaction (p=0.037), STAI State (p<0.001), DASS-21 (p=0.001), PHQ (p<0.001), OCI-R (p=0.002) and PSS (p<0.001). Among the matched individuals who answered the survey in May 2020 (n=93), we found that the group who maintained follow-up (n=24) did not improve significantly more than the other group (n=69) for any of the mental health variables in study.

**Conclusions:**

The results indicate that stopping psychiatric and psychological follow-up represented worse mental health outcomes at the beginning of the first confinement. However, anxiety feelings improved at the end of the first confinement, which happened independently of psychiatric/ psychological follow-up.

**Disclosure of Interest:**

None Declared

